# Serum Liver Fibrosis Markers for Predicting the Presence of Gastroesophageal Varices in Liver Cirrhosis: A Retrospective Cross-Sectional Study

**DOI:** 10.1155/2015/274534

**Published:** 2015-12-06

**Authors:** Xingshun Qi, Hongyu Li, Jiang Chen, Chunlian Xia, Ying Peng, Junna Dai, Yue Hou, Han Deng, Jing Li, Xiaozhong Guo

**Affiliations:** Liver Cirrhosis Study Group, Department of Gastroenterology, General Hospital of Shenyang Military Area, 83 Wenhua Road, Shenyang 110840, China

## Abstract

*Background and Aims*. A retrospective cross-sectional study was conducted to evaluate the role of hyaluronic acid (HA), laminin (LN), amino-terminal propeptide of type III procollagen (PIIINP), and collagen IV (CIV) in predicting the presence of gastroesophageal varices (GEVs) in patients with liver cirrhosis.* Methods*. We enrolled 118 patients with liver cirrhosis who underwent the tests for the four serum liver fibrosis markers and upper gastrointestinal endoscopy at the same admissions. The predictive values of the four serum liver fibrosis markers were evaluated by the areas under the receiving operator characteristics curves (AUROCs) with 95% confidence intervals (CIs).* Results*. The prevalence of GEVs was 88% (104/118). The AUROCs for HA, LN, PIIINP, and CIV levels in predicting the presence of GEVs were 0.553 (95% CI: 0.458 to 0.644, *P* = 0.5668), 0.490 (95% CI: 0.397 to 0.584, *P* = 0.9065), 0.622 (95% CI: 0.528 to 0.710, *P* = 0.1099), and 0.560 (95% CI: 0.466 to 0.652, *P* = 0.4909). The PIIINP level at a cut-off value of 31.25 had a sensitivity of 73.1% and a specificity of 57.1%.* Conclusions*. The present study did not recommend HA, LN, PIIINP, and CIV levels to evaluate the presence of GEVs in liver cirrhosis.

## 1. Introduction

Variceal bleeding is the most common life-threatening complication of liver cirrhosis [1]. Routine follow-up surveillance for gastroesophageal varices (GEVs) in such patients is important for establishing an early diagnosis and adopting the preventive measures. Upper gastrointestinal endoscopy is the golden diagnostic method for GEVs in our everyday clinical practice. According to the current consensus and practice guidelines recommendations, once liver cirrhosis is diagnosed, the patients should undergo the screening endoscopy for assessing GEVs [2, 3]. If the varices are lacking, the patients should undergo the endoscopy every 2-3 years, and if the varices are at a low risk, they should undergo the endoscopy every 1-2 years. But the endoscopy has its limitations, such as invasiveness, relatively high cost, procedure-related complications, poor patients' compliance, and high demand of endoscopists' skills. The limitations become more obvious in patients who need repeated endoscopy. Nowadays, noninvasive diagnostic tests of GEVs have been widely explored [4, 5]. They primarily include the liver and spleen stiffness measurement [6–10], platelet count/spleen diameter ratio [11, 12], aspartate aminotransferase/platelets count ratio [13, 14], portal venous haemodynamic parameters [15], and indocyanine green “15-minute” retention test, or other liver function tests [16]. Indeed, these noninvasive markers for the diagnosis of GEVs are also frequently used to reflect the severity of liver fibrosis [17, 18]. Thus, it appears to be reasonable to assume that hyaluronic acid (HA), laminin (LN), amino-terminal propeptide of type III procollagen (PIIINP), and collagen IV (CIV), which are four major serum markers for liver fibrosis, are also eligible for the assessment of GEVs.

Until now, few studies have evaluated the role of serum liver fibrosis markers in predicting the presence of GEVs in patients with liver cirrhosis. Gressner et al. evaluated the correlation of LN and PIIINP concentrations with portal venous pressure in patients with liver fibrosis (*n* = 21) and cirrhosis (*n* = 12) [19]. They found a positive correlation between LN and portal venous pressure. The same study team further confirmed the diagnostic accuracy of HA and LN in the assessment of portal hypertension [20]. Kondo et al. also found a positive and significant correlation between LN and hepatic vein pressure gradient in 20 patients with alcoholic liver cirrhosis [21]. However, these investigators suggested that LN should not be a reliable marker for assessing the risk of variceal bleeding. Generally, the limited evidence originates from old studies with small sample sizes and heterogeneous study population. Herein, we conducted a retrospective cross-sectional study to evaluate the predictive values of serum liver fibrosis markers (i.e., HA, LN, PIIINP, and CIV) for the presence of GEVs.

## 2. Methods

### 2.1. Study Design

In this study, the eligibility criteria were as follows: (1) patients were admitted to the General Hospital of Shenyang Military Area between January 2013 and June 2014; (2) patients were diagnosed with liver cirrhosis; (3) patients underwent the tests for the four serum liver fibrosis markers (i.e., HA, LN, PIIINP, and CIV) at their admissions; and (4) patients underwent upper gastrointestinal endoscopy to evaluate the presence of varices at the same admissions. This study was conceived by two investigators (Qi X. and Guo X.), and the study protocol was approved by the Medical Ethical Committee of the General Hospital of Shenyang Military Area (number k(2015)03).

Electronic medical charts were retrospectively reviewed. The following data were collected: age, sex, presence of malignancy, etiology of liver cirrhosis, clinical presentation, red blood cell (RBC), hemoglobin (Hb), white blood cell (WBC), platelets count (PLT), total bilirubin (TBIL), albumin (ALB), alanine aminotransferase (ALT), aspartate aminotransferase (AST), alkaline phosphatase (ALP), gamma-glutamine transferase (GGT), blood urea nitrogen (BUN), creatinine (Cr), prothrombin time (PT), activated partial thromboplastin time (APTT), international normalized ratio (INR), severity of liver dysfunction (Child-Pugh and model for the end-stage of liver diseases score), and HA, LN, PIIINP, and CIV levels. The laboratory tests for the HA, LN, PIIINP, and CIV were performed by using the chemiluminescent immunoassay in the LUmo Microplate Luminometer equipment. The diagnostic kits for the HA, LN, PIIINP, and CIV were also purchased from the Autobio Diagnostics Co., Ltd. (Zhengzhou, Henan Province, China). The reference values were as follows: HA < 120 ng/mL, LN < 130 ng/mL, PIIINP < 15 ng/mL, and CIV < 95 ng/mL. Other laboratory tests were performed in the Department of Clinical Laboratory of our hospital.

Severity of EVs was classified into no, mild, and moderate/severe according to the 2008 Hangzhou consensus. It was jointly published in Chinese language by the Chinese Society of Gastroenterology, Chinese Society of Hepatology, and Chinese Society of Digestive Endoscopy. Briefly, if there were red color signs, the varices were considered as moderate/severe. Otherwise, the forms of varices were further identified. If the forms were linear, they were considered as mild. If the forms were snake-like, they were considered as moderate. If the forms were bead-like or tubercular, they were considered as severe.

### 2.2. Statistical Analyses

Categorical and continuous variables were reported as the frequencies and means ± standard errors, respectively. Continuous variables were compared by the independent sample *t*-tests. Receiving operator characteristics (ROC) curve analysis was performed to identify the discriminative capacity of HA, LN, PIIINP, and CIV levels in predicting the presence of GEVs. A cut-off value of HA, LN, PIIINP, or CIV level was chosen as both sensitivity and specificity were optimal. Areas under the ROC curves (AUROCs) with 95% confidence intervals (CIs) were also reported. *P* < 0.05 was considered statistically significant. All statistical analyses were performed by using the MedCalc software version 11.4.2.0 and SPSS Statistics version 17.0.0.

## 3. Results

During this period, a total of 272 patients with liver cirrhosis underwent the tests for the HA, LN, PIIINP, and CIV levels. Notably, in our previous study, 228 of them had been enrolled to evaluate the correlation of the four serum liver fibrosis markers with the severity of liver dysfunction [22]. In the present study, 118 of them were enrolled to evaluate the correlation of serum liver fibrosis markers with the presence of GEVs (Table 1), because 154 patients did not undergo upper gastrointestinal endoscopy at admissions. Among these included patients, 10 had a diagnosis of hepatocellular carcinoma, and 3 had previous history of other malignancy (thyroid carcinoma *n* = 1, renal carcinoma *n* = 1, and rectal cancer *n* = 1). The major causes of hospitalization included upper gastrointestinal bleeding (*n* = 69), weakness (*n* = 18), massive ascites (*n* = 10), abdominal distension (*n* = 6), abdominal pain (*n* = 6), jaundice (*n* = 4), incident diagnosis of liver cirrhosis (*n* = 3), hepatic encephalopathy (*n* = 1), and gingival bleeding (*n* = 1). The etiology of liver cirrhosis included hepatitis B virus infection alone (*n* = 41), hepatitis C virus infection alone (*n* = 11), a combination of hepatitis B virus and hepatitis C virus infections (*n* = 1), alcohol alone (*n* = 27), a combination of hepatitis B virus and alcohol (*n* = 4), autoimmune hepatitis (*n* = 3), drug-related (*n* = 5), and unknown (*n* = 26). The prevalence of GEVs was 88% (104/118). GEVs were severe, moderate, and mild in 46, 21, and 16 patients, respectively. Notably, the severity of GEVs was unclear in 21 patients. The location of GEVs included esophageal varices alone (*n* = 48), esophageal and gastric varices (*n* = 52), and gastric varices alone (*n* = 4).

**Figure 1:**
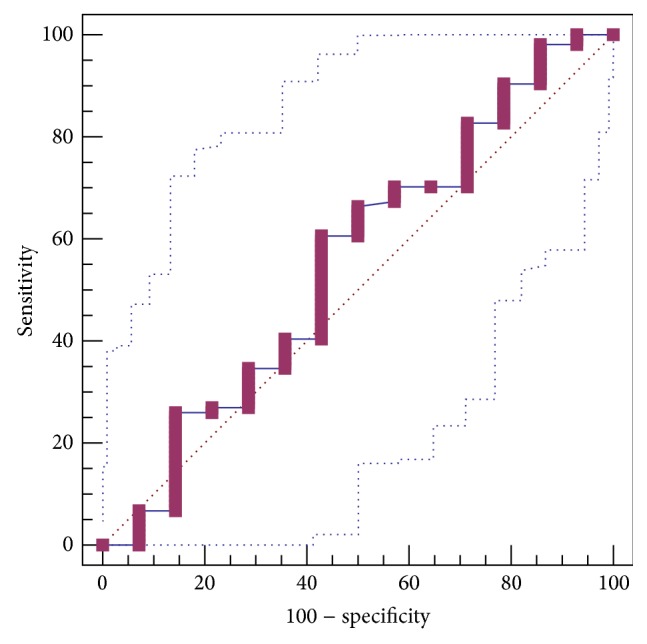
ROC curve analysis to identify the discriminative capacity of HA level in predicting the presence of GEVs in liver cirrhosis.

HA level was not significantly different between patients with and without GEVs (621.21 ± 117.21 versus 1511.77 ± 1137.21). The AUROC for HA level in predicting the presence of GEVs was 0.553 ± 0.0917 (95% CI: 0.458 to 0.644, *P* = 0.5668) (Figure 1). The best cut-off value was 299.24 with a sensitivity of 60.6% and a specificity of 57.1%.

**Figure 2:**
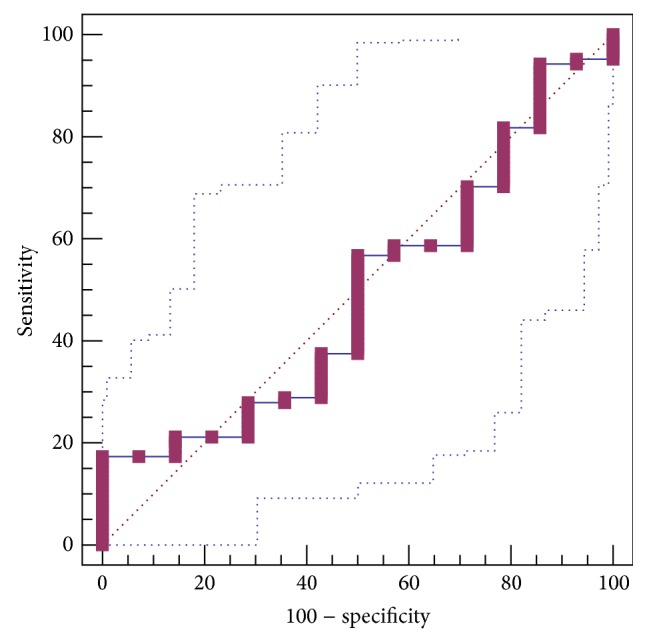
ROC curve analysis to identify the discriminative capacity of LN level in predicting the presence of GEVs in liver cirrhosis.

LN level was not significantly different between patients with and without GEVs (142.86 ± 10.31 versus 133.60 ± 23.84). The AUROC for LN level in predicting the presence of GEVs was 0.490 ± 0.0818 (95% CI: 0.397 to 0.584, *P* = 0.9065) (Figure 2). The best cut-off value was 39.73 with a sensitivity of 17.3% and a specificity of 100%.

**Figure 3:**
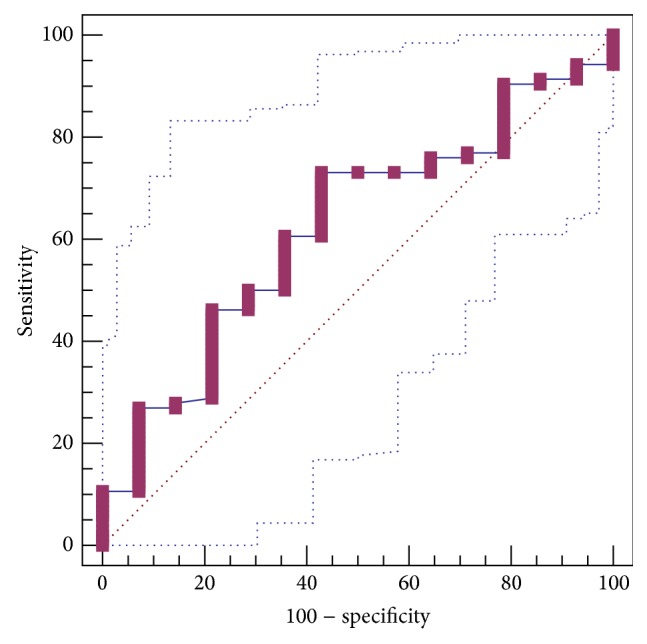
ROC curve analysis to identify the discriminative capacity of PIIINP level in predicting the presence of GEVs in liver cirrhosis.

PIIINP level was not significantly different between patients with and without GEVs (93.76 ± 12.00 versus 53.16 ± 13.48). The AUROC for PIIINP level in predicting the presence of GEVs was 0.622 ± 0.0762 (95% CI: 0.528 to 0.710, *P* = 0.1099) (Figure 3). The best cut-off value was 31.25 with a sensitivity of 73.1% and a specificity of 57.1%.

**Figure 4:**
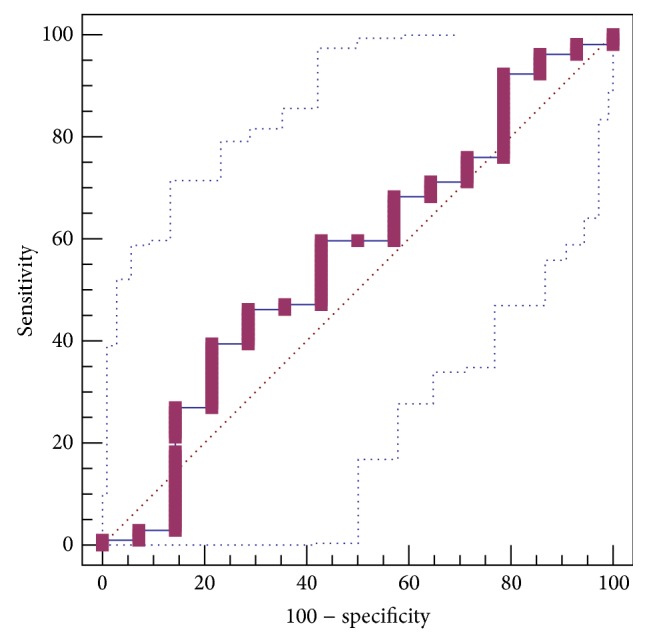
ROC curve analysis to identify the discriminative capacity of CIV level in predicting the presence of GEVs in liver cirrhosis.

CIV level was not significantly different between patients with and without GEVs (153.12 ± 14.47 versus 188.51 ± 48.58). The AUROC for CIV level in predicting the presence of GEVs was 0.560 ± 0.0877 (95% CI: 0.466 to 0.652, *P* = 0.4909) (Figure 4). The best cut-off value was 95.3 with a sensitivity of 39.4% and a specificity of 78.6%.

## 4. Discussion

The liver fibrosis progression is the major cause for the occurrence and development of portal hypertension in liver cirrhosis. GEVs are one of the most important portal hypertension-related complications. It is reasonable to suppose that the markers of liver fibrosis may reflect the presence and severity of GEVs. However, we would like to emphasize that only very few studies have evaluated the correlation between serum liver fibrosis markers and varices in liver cirrhosis [19–21]. By comparison, the role of liver stiffness measurement in predicting varices in liver cirrhosis has been widely discussed. Herein, we attempted to indirectly discuss the possible role of noninvasive diagnostic methods of liver fibrosis by presenting the data from liver stiffness measurement.

Numerous studies and meta-analyses confirmed the significance of liver stiffness measurement for the diagnosis of liver fibrosis. Friedrich-Rust et al. performed a meta-analysis of 50 studies to assess the performance of transient elastography for diagnosing with liver fibrosis [23]. They found that transient elastography carried an excellent diagnostic accuracy with a mean AUROC of 0.84 (95% CI: 0.82 to 0.86) for significant fibrosis, 0.89 (95% CI: 0.88 to 0.91) for severe fibrosis, and 0.94 (95% CI: 0.93 to 0.95) for cirrhosis. Chon et al. also conducted a meta-analysis of 18 studies to evaluate the performance of transient elastography for diagnosing with liver fibrosis in patients with chronic hepatitis B virus infection [24]. Similarly, transient elastography provided a good diagnostic accuracy with a mean AUROC of 0.859 (95% CI: 0.857 to 0.860) for significant fibrosis, 0.887 (95% CI: 0.886 to 0.887) for severe fibrosis, and 0.929 (95% CI: 0.928 to 0.929) for cirrhosis. More recently, Singh et al. performed a meta-analysis of individual participant data from 12 studies and found that magnetic resonance elastography had a high diagnostic accuracy with a mean AUROC of 0.84 (95% CI: 0.76 to 0.92) for any fibrosis, 0.88 (95% CI: 0.84 to 0.91) for significant fibrosis, 0.93 (95% CI: 0.90 to 0.95) for severe fibrosis, and 0.92 (95% CI: 0.90 to 0.94) for cirrhosis [25].

On the other hand, there was a good correlation of liver stiffness with hepatic venous pressure gradient and varices in liver cirrhosis [5, 10, 18, 26]. In the study by Castéra et al., the sensitivity and specificity of transient elastography at a cut-off value of 21.5 kPa in predicting the presence of varices were 76% and 78%, respectively [6]. In the study by Saad et al., liver stiffness measured by FibroScan was regarded as the independent predictor of GEVs (odds ratio = 1.113, *P* = 0.005) [7]. In addition, compared with hepatic venous pressure gradient, liver stiffness also had a similar performance for predicting the occurrence of portal hypertension-related complications (AUROC: 0.815 (95% CI: 0.727 to 0.903) versus 0.837 (95% CI: 0.754 to 0.920)) [27].

In accordance with such a good performance of the liver stiffness measurement in assessing both liver fibrosis and GEVs, further studies may be attractive to evaluate the potential utility of serum liver fibrosis markers tests. Indeed, the clinical evidence has demonstrated that serum liver fibrosis markers are useful in diagnosing the presence of liver fibrosis and classifying its severity [28, 29]. However, it remains unclear whether or not serum liver fibrosis markers can be also useful for screening the presence of GEVs.

Our study did not confirm any significant predictive values of HA, LN, PIIINP, and CIV levels in the assessment of GEVs in liver cirrhosis. This unexpected finding could be explained by a high proportion of patients with history of upper gastrointestinal bleeding and a small number of patients without GEVs (*n* = 14), which might result in the instability of their statistical results. The imbalance in the number of observed patients between the two groups might be primarily attributed to the bias of patient selection. Indeed, only in-patients were enrolled in our study, suggesting that most of our cases had relatively severe conditions or were at decompensated stage. Additionally, serum liver fibrosis markers are more likely to reflect the fibrogenesis, rather than the status of fibrosis. They correlate with the activity of liver diseases (i.e., active alcohol abuse or viral replication). Serum liver fibrosis markers become normal, if liver disease is inactive. Accordingly, it may be less likely to be in a good correlation of serum liver fibrosis markers with portal hypertension.

In spite of the fact that there is no statistical significance, the detailed information should be mentioned. (1) PIIINP level was higher in patients with GEVs than in those without. (2) The mean AUROC for PIIINP was 0.622. (3) *P* value was close to 0.05. Thus, well-designed studies with appropriate selection of out-patients or patients at compensated stage should validate the role of PIIINP in predicting the presence of GEVs in liver cirrhosis.

In conclusion, based on this retrospective study, the HA, LN, PIIINP, and CIV levels cannot be recommended to evaluate the presence of GEVs in liver cirrhosis. Certainly, the potential utility of PIIINP should be further confirmed in prospective studies. Additionally, given the retrospective nature of this study, the severity and location of GEVs were not clearly evaluated in all patients according to the medical records and endoscopy reports. Accordingly, further studies might be warranted to identify the role of serum liver fibrosis in predicting the presence of large varices or gastric/esophageal varices in liver cirrhosis.

## Figures and Tables

**Table 1:** Characteristics of patients.

Variables	Values
Age (years)	55.14 ± 0.98
Sex (male/female): *n*	76/42
Hepatocellular carcinoma (yes/no): *n*	10/108
Ascites (yes/no); *n*.	52/66
Hepatic encephalopathy (yes/no): *n*	2/116
Gastroesophageal varices (yes/no): *n*	104/14
Red blood cell (10^12^/L)	3.15 ± 0.07
Hemoglobin (g/L)	89.68 ± 2.52
White blood cell (10^9^/L)	4.16 ± 0.24
Platelets count (10^9^/L)	91.11 ± 5.71
Total bilirubin (umol/L)	26.90 ± 2.37
Albumin (g/L)	32.92 ± 0.58
Alanine aminotransferase (U/L)	46.91 ± 10.91
Aspartate aminotransferase (U/L)	60.64 ± 7.43
Alkaline phosphatase (U/L)	108.12 ± 6.93
Gamma-glutamine transferase (U/L)	134.29 ± 40.40
Blood urea nitrogen (mmol/L)	6.01 ± 0.32
Creatinine (umol/L)	63.72 ± 3.06
Prothrombin time (seconds)	16.00 ± 0.25
Activated partial thromboplastin time (seconds)	42.05 ± 0.63
International normalized ratio	1.29 ± 0.03
Child-Pugh score	7.09 ± 0.17
Model for the end-stage of liver diseases score	5.59 ± 0.47
Hyaluronic acid (ng/mL)	726.87 ± 168.58
Laminin (ng/mL)	141.76 ± 9.49
Amino-terminal propeptide of type III procollagen (ng/mL)	88.94 ± 10.75
Collagen IV (ng/mL)	157.32 ± 13.95
Hyaluronic acid above the reference value: *n*.	117
Laminin above the reference value: *n*	71
Amino-terminal propeptide of type III procollagen above the reference value: *n*	114
Collagen IV above the reference value: *n*	83
